# Profiles of humanistic practice ability in psychiatric nurses: a latent profile analysis and associated factors

**DOI:** 10.3389/fpsyt.2026.1898793

**Published:** 2026-09-14

**Authors:** Yi Chen, Minzhi Sun, Guowei Chen

**Affiliations:** 1 School of Medicine and Nursing, Nanjing University of Chinese Medicine Hanlin College, Nanjing University of Chinese Medicine, Nanjing, China; 2 College of Basic Medicine and Nursing, Zhongshan Vocational College, Nanjing, China; 3 Department of Pediatrics, Zhongda Hospital, Southeast University, Nanjing, China

**Keywords:** emotional intelligence, humanistic practice ability, job stressors, latent profile analysis, psychiatric nurses

## Abstract

**Objectives:**

To identify latent profiles of humanistic practice ability among psychiatric nurses using latent profile analysis and to examine factors associated with profile classification.

**Methods:**

A cross-sectional survey was conducted on the humanistic practice ability of 639 psychiatric nurses using a convenience sampling method. Latent profile analysis and multivariate logistic regression were employed to analyze the data.

**Results:**

Psychiatric nurses’ humanistic practice ability was classified into three latent profiles, Low-Level Humanistic Practice Profile (29.9%), Moderate-Level Humanistic Practice Profile (57.3%), High-Level Humanistic Practice Profile (12.8%). Years of working, emotional intelligence, and job stressors were significantly associated with classification in different latent profiles (P < 0.05).

**Conclusions:**

This study revealed the latent heterogeneity in humanistic practice ability among psychiatric nurses and identified individual and work environment factors associated with different profile membership. The findings can help nursing managers identify subgroups of nurses with distinct characteristics of humanistic practice ability, and provide a basis for future stratified assessment as well as the design and evaluation of differentiated support strategies.

## 1 Introduction

Nurses’ humanistic practice ability refers to the integration of humanistic values into clinical practice through specific techniques, procedures, and approaches. It reflects a nurse’s ethical literacy, professional knowledge, interpersonal skills, and overall mental outlook (1, 2). Nurses with good humanistic practice ability are better able to understand patients’ emotional and psychosocial needs and to provide patient-centered care (3, 4). This ability is important in psychiatric nursing, as psychiatric nurses must address patients’ complex psychological needs, abnormal emotional fluctuations, potential risks of violence or self-harm, and high demands for emotional labor (5–7). A higher level of humanistic practice ability may help nurses establish trusting nurse-patient relationships, and may be associated with a greater sense of professional achievement (8, 9) and a lower risk of professional burnout and emotional exhaustion (10). However, current research on nurses’ humanistic practice ability has mainly focused on nurses in general hospitals and newly graduated nurses (11, 12), with relatively limited research on psychiatric nurses as a distinct group.

Psychiatric nurses face high workloads, safety risks, complex needs of patients and their families, and occupational stress in their clinical work, and these work environment factors may affect their clinical nursing practice (13, 14). A previous study on ICU nurses found that higher levels of nurses’ job stressors were associated with lower humanistic practice ability (15). However, relevant research on psychiatric nurses remains limited, and the relationship between job stressors and membership in different latent profiles of humanistic practice ability remains unclear. Emotional intelligence (EI) refers to the ability to understand, identify, regulate, and use one’s own and others’ emotions (6), and may help nurses maintain effective communication and emotional regulation in high-stress clinical situations (16–18). Similarly, whether and how EI is associated with membership in different latent profiles of humanistic practice ability among psychiatric nurses remains unclear.

This study adopted the Triadic Interaction Theory as its theoretical framework. This theory emphasizes the interrelationships among individual factors, environment factors, and behavioral performance (19). In this study, EI was regarded as an individual-level factor, nurses’ job stressors were regarded as an environment factor, and humanistic practice ability was regarded as a behavioral outcome related to nursing practice. This framework provides a theoretical basis for considering individual and environmental factors together when examining heterogeneity in psychiatric nurses’ humanistic practice ability.

The previous study has generally used total scale scores or single-dimension scores to evaluate nurses’ humanistic practice ability, such a variable-centered approach may obscure the heterogeneity of different ability patterns within the nurse population. Latent profile analysis (LPA) is a person-centered classification statistical method that can identify individuals’ latent characteristics based on their response patterns on manifest variables, grouping individuals with similar characteristics into the same profile and calculating the probability of profile membership (20). Therefore, this study aimed to: (1) identify latent profiles of humanistic practice ability among psychiatric nurses using LPA; (2) compare sociodemographic and occupational characteristics across the different latent profiles; and (3) examine the associations of EI and nurses’ job stressors with latent profile membership. By identifying subgroups that may not be captured by traditional total-score analyses, this study is expected to enhance the understanding of heterogeneity in humanistic practice ability among psychiatric nurses and provide a basis for subsequent stratified assessment and further investigation of factors associated with different profiles.

## 2 Methods

### 2.1 Participants and sample size

From February to May 2025, clinical psychiatric nurses from psychiatric hospitals in Jiangsu Province were recruited using convenience sampling. Participants were drawn from different types of psychiatric nursing units, including acute wards, chronic wards, and rehabilitation wards, in order to increase the diversity of the sample’s clinical background.

1. Inclusion criteria were:
  - clinical psychiatric nurses providing direct patient care;
  - at least 1 year of clinical nursing experience;
  - holding a valid registered nurse license.
2. Exclusion criteria included:
  - nursing interns and nurses undergoing temporary training;
  - nurses who were on leave or attending training outside the hospital during the data collection period.

There is no universally established fixed sample-size criterion for LPA, as sample-size requirements depend on factors such as profile separation, the number of latent profiles, the number of observed indicators, and sample size itself (21). Previous simulation study of LPA and mixture models further demonstrate that sample-size adequacy should be evaluated in conjunction with specific model characteristics (22). A total of 715 questionnaires were collected, of which 639 were valid, yielding a valid response rate of 89.37%. With the five Humanistic Practice Ability of Nursing (HPAN) dimensions used as observed indicators, the final analytic sample of 639 participants provided a substantial sample for the planned LPA.

### 2.2 Ethics

The study was conducted in accordance with the principles of the Declaration of Helsinki and was reviewed and approved by the Institutional Review Board of Zhongda Hospital Southeast University (2024ZDSYLL261-P01). All the data collected from the subjects were kept anonymous and confidential to protect the privacy of the study subjects.

### 2.3 Data collection and quality control

Data were collected through the online platform Wenjuanxing. A questionnaire link (QR code) was distributed via WeChat to a network of psychiatric nurses. The first page of the questionnaire provided standardized study information, including the purpose and significance of the study, instructions for completion, and informed consent information. To reduce duplicate responses and improve the completeness of the questionnaire data, the online questionnaire could only be accessed by scanning a QR code via WeChat. Each WeChat account was allowed to respond only once, and a resume-from-break function was enabled. To verify eligibility, respondents were asked, “Are you involved in the clinical care of psychiatric patients?” A “No” response led to automatic disqualification and page closure. All questions were mandatory, with automated prompts for missing items.

### 2.4 Measurements

The general information questionnaire included hospital level, gender, age, years of working, professional position, Educational level, employment forms, night shift, education training of humanistic nursing, and undertake teaching task.

The Humanistic Practice Ability of Nursing (HPAN) Scale, developed by Yan Haiping (23), was used to assess the psychiatric nurses’ humanistic practice ability. This scale comprises 26 items across 5 dimensions, humanistic care practice, interpersonal communication, self-management, psychological adjustment, and ethics and legal practice. The 26 items are rated on a 5-point Likert scale (1 = strongly disagree, 5 = strongly agree). Higher scores indicate higher ability. The Cronbach’s alpha coefficient of the scale was 0.913 (23). The Cronbach’s alpha in this study was 0.880.

Wong and Law Emotional Intelligence Scale (WLEIS-C) was developed by Wong and Law in 2002 (24) and later translated into Chinese, revised, and validated by Wang Yefei (25). The Chinese version was used in this study to measure nurses’ EI across 4 dimensions, self-emotion appraisal (SEA), others’ emotion appraisal (OEA), regulation of emotion (ROE), and use of emotion (UOE). It consists of 16 items rated on a 7-point Likert scale (0 = strongly disagree, 6 = strongly agree). Higher scores reflect stronger EI. The Cronbach’s alpha coefficient of the Chinese version of the scale was 0.902 (25). The Cronbach’s alpha for this study was 0.915.

Chinese Nurse Job Stressors Scale, revised by Wang Yu et al. (26) to measure nurses’ perceived job stressors across 5 dimensions, work environment and character, doctor-nurse relationship, the attitude of patients and families, professional skill, and social status and career development. It consists of 38 items rated on a 4-point Likert scale (0 = no stress, 3 = high stress). Higher scores represent greater perceived stress. The Cronbach’s alpha coefficient of the scale was 0.957 (26). The Cronbach’s alpha was 0.906.

### 2.5 Statistical analysis

LPA was conducted using Mplus 8.3, with the 5 HPAN subscales as observed variables. Model fit was assessed using Akaike Information Criterion (AIC), Bayesian Information Criterion (BIC), and sample-size adjusted BIC (aBIC). Lower values indicate better fit. Entropy values >0.8 indicated classification accuracy >90% (27). The Lo-Mendell-Rubin adjusted likelihood ratio test (LMRT) and bootstrap likelihood ratio test (BLRT) were used for model comparison. A p-value <0.05 suggested that a k-class model significantly outperformed a k–1 model. Models were also evaluated for classification probability and subgroup size, with acceptable profiles exceeding 5% of the sample.

Second, SPSS 27.0 software was used for data analysis. Categorical data were described using frequencies and percentages, while continuous variables were presented as means and standard deviations ( $\bar{x}\pm s$). Comparisons between categorical variables were performed using the chi-square (χ^2^) test or Fisher’s exact test, as appropriate. Finally, multivariate logistic regression was conducted, using LPA as the dependent variable and statistically significant predictors from univariate analysis as independent variables. Multicollinearity among the independent variables included in the multinomial logistic regression model was assessed using tolerance and variance inflation factor (VIF).A p-value <0.05 was considered statistically significant.

## 3 Results

### 3.1 Demographic characteristics of participants

As shown in **Table 1**, a total of 639 psychiatric nurses participated in this study. Of these, 65.26% were employed at Grade 3 hospitals, and 84.04% were female, which is significantly higher than the proportion of male nurses. Nurses aged between 30 and 39 accounted for 48.20%, while 45.70% of nurses had less than 10 years of working. Regarding professional position, the largest proportion (47.89%) were nurse practitioner, followed by nurse supervisor and above, which accounted for 37.72%. The highest proportion of nurses (80.44%) were undergraduate and above. Non-formal employed nurses made up the largest group at 70.11%. In clinical work, 79.03% of the nurses worked night shift. Among the psychiatric nurses surveyed, 35.21% undertook teaching tasks, and 67.61% had received education training of humanistic nursing.

**Table 1:** Sociodemographic and occupational characteristics of participants (N = 639).

Variable	n (%)
Gender	
Male	102(15.96)
Female	537(84.04)
Hospital level	
Grade 3	417(65.26)
Grade 2 and below	222(34.74)
Age (years)	
≥40	87(13.62)
30-39	308(48.20)
<30	244(38.18)
Years of working	
≥20	75(11.74)
10-19	272(42.56)
<10	292(45.70)
Professional position	
Nurse supervisor and above	241(37.72)
Nurse Practitioner	306(47.89)
Nurse	92(14.40)
Educational level	
Undergraduate and above	514(80.44)
College degree and below	125(19.56)
Employment forms	
Formal employed	191(29.89)
Non-formal employed	448(70.11)
Night shift	
No	134(20.97)
Yes	505(79.03)
Education training of humanistic nursing	
Yes	432(67.61)
No	207(32.39)
Undertake teaching tasks	
Yes	225(35.21)
No	414(64.79)

### 3.2 LPA of humanistic practice ability

LPA was conducted using the 5 subscales of the HPAN as observed indicators, testing models with 1 to 4 latent classes. As shown in **Table 2**, with increasing class numbers, the entropy values exceeded 0.8 and AIC, BIC, and aBIC values decreased, indicating better model fit. Although the four-class model had higher entropy, it included a class with only 1.7% of the sample, suggesting poor class separation. Therefore, the three-class model was selected based on both statistical and theoretical considerations. The average posterior probabilities of classification were 98.7%, 97.0%, and 94.7%, indicating good classification quality.

**Table 2:** Results and naming of latent profile analysis of psychiatric nurses’ humanistic practice ability.

Model	Log(L)	AIC	BIC	aBIC	Entropy	*P*		Classification probability			
						LMRT	BLRT				
1	-7765.663	15551.325	15595.924	15564.175	—	—	—	1			
2	-6691.556	13415.113	13486.471	13435.673	0.947	<0.001	<0.001	0.341	0.659		
3	-6373.939	12791.879	12889.996	12820.148	0.941	0.0043	<0.001	0.299	0.573	0.128	
4	-6104.004	12264.008	12388.885	12299.987	0.958	<0.001	<0.001	0.017	0.131	0.554	0.297

AIC, Akaike information criterion; BIC, Bayesian information criterion; aBIC, sample-size-adjusted Bayesian information criterion; LMRT, Lo-Mendell-Rubin adjusted likelihood ratio test; BLRT, Bootstrap likelihood ratio test.

**Figure 1** illustrates the latent profiles based on mean scores across the five HPAN dimensions. All latent profiles showed a consistent gradient pattern across the five dimensions, with no substantial differences in dimensional structure observed. Therefore, profiles were named based on overall levels, using the descriptors “low,” “moderate,” and “high” humanistic practice ability. Comparison of the three latent profiles showed that C1 had the lowest overall scores (77.356 ± 7.576) and were classified as the Low-Level Humanistic Practice Profile, comprising 191 nurses (29.9%). C2 had intermediate scores (100.202 ± 5.254), falling between C1 and C3, and was defined as Moderate-Level Humanistic Practice Profile, including 366 nurses (57.3%). C3 had the highest overall scores (116.073 ± 5.337) and was classified as High-Level Humanistic Practice Profile, comprising 82 nurses (12.8%). One-way ANOVA showed significant differences in humanistic practice ability scores across the three groups (F = 1203.547, P < 0.001), as shown in **Table 3**.

**Figure 1:**
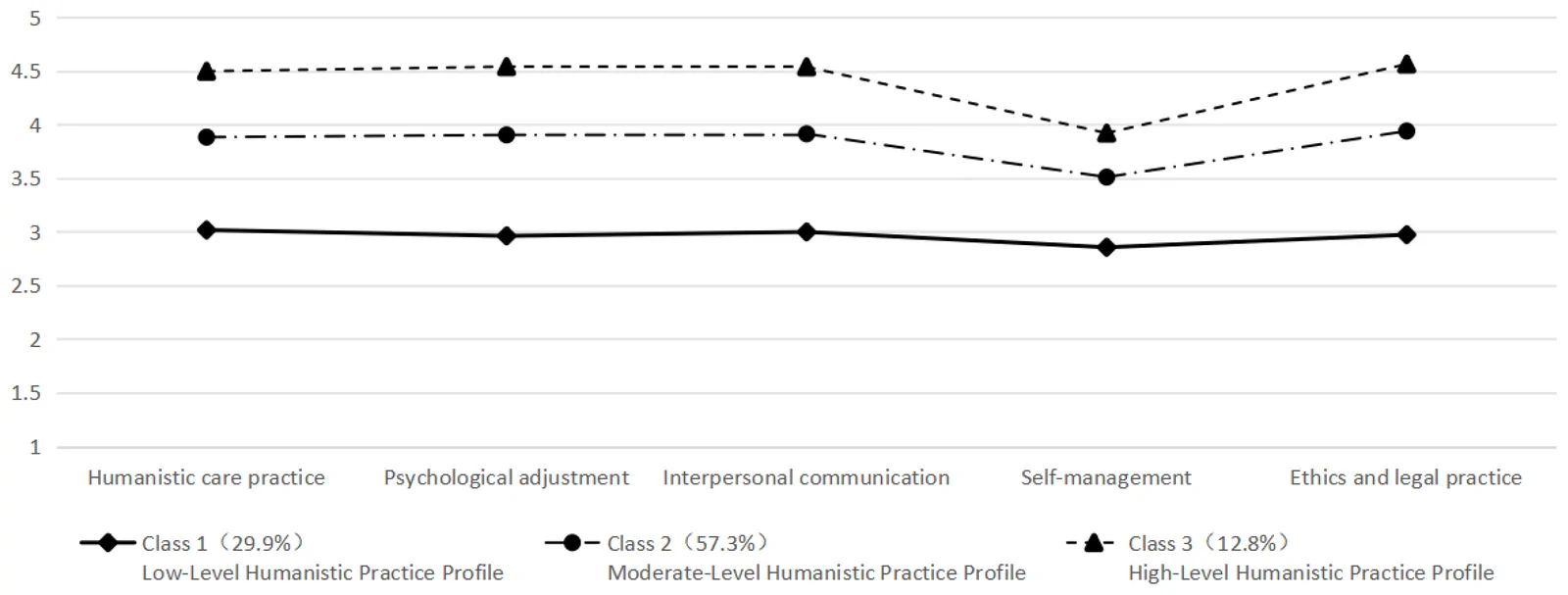
Latent profiles of humanistic practice ability among psychiatric nurses based on mean scores across the five HPAN dimensions. The x-axis represents the five dimensions of the Humanistic Practice Ability of Nursing (HPAN) scale: humanistic care practice, psychological adjustment, interpersonal communication, self-management, and ethics and legal practice. The y-axis represents the mean item score for each HPAN dimension within each latent profile, with higher scores indicating higher levels of humanistic practice ability. C1 represents the Low-Level Humanistic Practice Profile (29.9%), C2 the Moderate-Level Humanistic Practice Profile (57.3%), and C3 the High-Level Humanistic Practice Profile (12.8%). The three profiles show a generally parallel graded pattern across the five dimensions.

**Table 3:** Comparison of scores of nurses’ humanistic practice ability in emotional intelligence ( $\bar{x}\pm s$).

Variable	Total scores	Low-level humanistic practice profile (C1,N=191)	Moderate-level humanistic practice profile (C2,N=366)	High-level humanistic practice profile (C3,N=82)	*F*	*P*
HPA of nurses	95.410 ± 14.217	77.356 ± 7.576	100.202 ± 5.254	116.073 ± 5.337	1203.547	<0.001
Humanistic care practice	37.011 ± 5.666	30.126 ± 3.402	38.809 ± 2.400	45.024 ± 2.885	772.460	<0.001
Psychological adjustment	14.808 ± 2.429	11.812 ± 1.316	15.607 ± 1.095	18.220 ± 1.078	975.627	<0.001
Interpersonal communication	22.310 ± 3.520	17.969 ± 1.997	23.462 ± 1.468	27.280 ± 1.759	859.278	<0.001
Self-management	10.095 ± 1.711	8.550 ± 1.238	10.519 ± 1.338	11.805 ± 1.409	225.109	<0.001
Ethics and legal practice	11.186 ± 1.906	8.901 ± 1.186	11.806 ± 0.908	13.744 ± 0.953	704.479	<0.001
Emotional intelligence	64.509 ± 12.752	53.12 ± 9.433	67.689 ± 10.681	76.768 ± 7.487	262.825	<0.001
Self-emotion appraisal	16.182 ± 3.515	13.361 ± 2.740	16.893 ± 3.067	19.573 ± 2.155	210.550	<0.001
Others’ emotion appraisal	16.316 ± 3.468	13.330 ± 2.628	17.172 ± 2.957	19.451 ± 2.195	221.410	<0.001
Use of emotion	15.942 ± 3.451	13.115 ± 2.874	16.795 ± 2.985	18.720 ± 2.086	176.610	<0.001
Regulation of emotion	16.069 ± 3.647	13.346 ± 2.975	16.828 ± 3.215	19.024 ± 2.914	131.932	<0.001
Nurse Job Stressors	52.285 ± 19.995	61.654 ± 21.947	49.500 ± 17.975	42.890 ± 15.227	37.402	<0.001
Work environment and character	17.236 ± 6.674	20.079 ± 7.339	16.284 ± 5.924	14.866 ± 6.126	28.476	<0.001
Doctor-nurse relationship	11.482 ± 6.561	14.921 ± 6.802	10.525 ± 5.960	7.744 ± 4.926	50.149	<0.001
Attitude of patients and their relatives	9.272 ± 3.702	10.277 ± 4.036	8.989 ± 3.477	8.195 ± 3.368	11.984	<0.001
Professional skills	7.784 ± 3.469	9.230 ± 3.641	7.413 ± 3.176	6.073 ± 3.118	31.402	<0.001
Social status and career development	6.510 ± 2.400	7.147 ± 2.559	6.290 ± 2.296	6.012 ± 2.197	10.322	<0.001

HPA, humanistic practice ability; C1, Low-Level Humanistic Practice Profile; C2, Moderate-Level Humanistic Practice Profile; C3, High-Level Humanistic Practice Profile;.

### 3.3 Comparison of HPAN, EI, and job stressors scores

As shown in **Table 3**, the theoretical score range of the scale was 26-130. In this study, the total HPAN score among psychiatric nurses was 95.410 ± 14.217. Subscale scores were as follows, humanistic care practice (37.011 ± 5.666), psychological adjustment (14.808 ± 2.429), interpersonal communication (22.310 ± 3.520), self-management (10.095 ± 1.711), and ethics and legal practice (11.186 ± 1.906). The total EI score was 64.509 ± 12.752. Subscale scores were as follows, SEA (16.182 ± 3.515), OEA (16.316 ± 3.468), UOE (15.942 ± 3.451), and ROE (16.069 ± 3.647). The total score for job stressors was 52.285 ± 19.995. Subscale scores were as follows, work environment and character (17.236 ± 6.674), doctor-nurse relationship (11.482 ± 6.561), patient and family attitudes (9.272 ± 3.702), professional skills (7.784 ± 3.469), and social status and career development (6.510 ± 2.400). Significant differences were observed among the three latent classes in terms of EI, job stressors, and all related subscales (P < 0.001).

### 3.4 Comparison of demographic data across latent profiles of nurses’ humanistic practice ability

Univariate analysis revealed significant differences across the three latent classes with respect to gender, hospital level, and years of working (see **Table 4**).

**Table 4:** Univariate comparison of demographic characteristics across latent profiles of humanistic practice ability (n=639) [*t*/*χ*^2^, n(%)].

Variable	Low-level humanistic practice profile (C1,N=191)	Moderate-level humanistic practice profile (C2,N=366)	High-level humanistic practice profile (C3,N=82)	*χ^2^*	*P*
Gender					
Male	42(21.99)	51(13.93)	9(10.98)	7.815	0.020
Female	149(78.01)	315(86.07)	73(89.02)		
Hospital level					
Grade 3	109(57.07)	244(66.67)	64(78.05)	11.888	0.003
Grade 2 and below	82(42.93)	122(33.33)	18(21.95)		
Age (years)					
≥40	20(10.47)	51(13.93)	16(19.51)	4.871	0.301
30-39	100(52.36)	171(46.72)	37(45.12)		
<30	71(37.17)	144(39.34)	29(35.37)		
Years of working					
≥20	16(8.38)	44(12.02)	15(18.29)	11.467	0.022
10-19	95(49.74)	141(38.52)	36(43.91)		
<10	80(41.88)	181(49.45)	31(37.80)		
Professional position					
Nurse supervisor and above	69(36.13)	131(35.79)	41(50.00)	8.342	0.080
Nurse Practitioner	88(46.07)	185(50.55)	33(40.24)		
Nurse	34(17.80)	50(13.66)	8(9.76)		
Educational level					
Undergraduate and above	151(79.06)	294(80.33)	69(84.15)	0.951	0.622
College degree and below	40(20.94)	72(19.67)	13(15.85)		
Employment forms					
Formal employed	56(29.32)	107(29.23)	28(34.15)	0.814	0.666
Non-formal employed	135(70.68)	259(70.77)	54(65.86)		
Night shift					
No	37(19.37)	79(21.58)	18(21.95)	0.425	0.808
Yes	154(80.63)	287(78.42)	64(78.05)		
Education training of humanistic nursing					
Yes	118(61.78)	258(70.49)	56(68.29)	4.370	0.113
No	73(38.22)	108(29.51)	26(31.71)		
Undertake teaching tasks					
Yes	70(036.65)	122(33.33)	33(40.24)	1.649	0.438
No	121(63.35)	244(66.67)	49(59.76)		

C1, Low-Level Humanistic Practice Profile; C2, Moderate-Level Humanistic Practice Profile; C3, High-Level Humanistic Practice Profile; χ^2^, chi-square statistic.

### 3.5 Logistic analysis of the factors influencing the latent profile of nurses’ humanistic practice ability

Variables with statistical significance in univariate analysis were included as independent variables, and latent profile was treated as the dependent variable in the multivariable logistic regression analysis. Values for each variable were assigned as follows, gender (male = 1, female = 2), hospital level (Grade 3 = 1, Grade 2 or below = 2), years of working (≥20 years as the reference group), EI and job stressors were entered as continuous variables. Multicollinearity diagnostics indicated no evidence of problematic multicollinearity among the independent variables, with tolerance values ranging from 0.364 to 0.951 and VIF values ranging from 1.052 to 2.749. The VIF values for EI and nurses’ job stressors were 1.125 and 1.191, respectively. The results of multivariable logistic regression analysis showed that years of working, EI, and job stressors were significantly associated with classification in different latent profiles (P < 0.05). Specifically, compared with the low-level profile (C1), nurses with higher EI scores were more frequently observed in the moderate-level (C2) and high-level (C3) profiles, whereas those with higher job stressors scores were more frequently observed in the low-level profile (C1). In addition, compared with nurses with ≥20 years of working, those with fewer years of working (<10 years and 10–19 years) were less frequently observed in the high-level profile (C3). Detailed regression coefficients and odds ratios are presented in **Table 5**.

**Table 5:** Logistic regression analysis of latent profile influencing factors of nurses’ humanistic practice ability.

Category	Variable	B	Standard error	Wald	*P*	Exp(B)	95% (*CI*)	
							Lower	Upper
Comparison between C1 and C2^*^	Intercept	-5.864	1.079	29.528	<0.001	—	—	—
	Female	0.393	0.303	1.691	0.193	1.482	0.819	2.682
	Grade 2 and below	-0.180	0.232	0.605	0.437	0.835	0.530	1.315
	Years of working <10 years	-0.302	0.394	0.588	0.443	0.739	0.341	1.601
	Years of working 10-19 years	-0.740	0.392	3.565	0.059	0.477	0.221	1.029
	Emotional intelligence	0.121	0.012	111.048	<0.001	1.129	1.104	1.155
	Nurse Job Stressors	-0.014	0.006	5.890	0.015	0.986	0.975	0.997
Comparison between C1 and C3^*^	Intercept	-13.538	1.956	47.888	<0.001	—	—	—
	Female	0.576	0.495	1.357	0.244	1.780	0.675	4.693
	Grade 2 and below	-0.596	0.381	2.445	0.118	0.551	0.261	1.163
	Years of working <10 years	-1.277	0.541	5.575	0.018	0.279	0.097	0.805
	Years of working 10-19 years	-1.280	0.532	5.788	0.016	0.278	0.098	0.789
	Emotional intelligence	0.230	0.021	118.087	<0.001	1.259	1.208	1.312
	Nurse Job Stressors	-0.034	0.010	11.331	0.001	0.966	0.947	0.986
Comparison between C2 and C3^★^	Intercept	-7.674	1.692	20.558	<0.001	—	—	—
	Female	0.183	0.414	0.195	0.658	1.201	0.534	2.702
	Grade 2 and below	-0.416	0.319	1.701	0.192	0.660	0.353	1.232
	Years of working <10 years	-0.975	0.398	5.998	0.014	0.377	0.173	0.823
	Years of working <10-19 years	-0.540	0.391	1.913	0.167	0.583	0.271	1.253
	Emotional intelligence	0.109	0.018	36.569	<0.001	1.115	1.076	1.155
	Nurse Job Stressors	-0.020	0.009	5.375	0.020	0.980	0.963	0.997

**B:** regression coefficient; C1: Low-Level Humanistic Practice Profile; C2: Moderate- Level Humanistic Practice Profile; C3: High-Level Humanistic Practice Profile; Years of working≥20 years was used as the reference category.; * Low-Level Humanistic Practice Profile is as reference; ☆ Moderate-Level Humanistic Practice Profile is as reference.

## 4 Discussion

In this study, the total score of humanistic practice ability among psychiatric nurses was 95.410 ± 14.217, which was lower than that reported in previous study of clinical nurses in China (12). Psychiatric nursing is characterized by strong interpersonal interaction and emotional labor, as nurses must continuously cope with patients’ complex psychological and behavioral problems as well as related safety risks in their work, and these occupational circumstances may increase their psychological and work burden (16). The previous study has also suggested that psychiatric nurses may face occupational stress issues related to sustained caregiving, such as compassion fatigue (28). Therefore, the findings of this study suggest that further attention should be paid to psychiatric nurses’ humanistic practice ability and its related work environment and individual factors. Further LPA revealed that psychiatric nurses’ humanistic practice ability exhibited different latent level patterns, and that years of working, EI, and nurses’ job stressors were associated with profile membership. These findings suggest that, in addition to attending to the overall level of humanistic practice ability among psychiatric nurses, attention should also be paid to individual differences within the group and their related factors.

This study used LPA to identify three latent profiles of humanistic practice ability: the Low-Level Profile, the Moderate-Level Profile, and the High-Level Profile. The three profiles showed a relatively consistent low-to-high gradient across the five dimensions of the HPAN. No distinct dimension-specific pattern was observed. This suggests that the differences among the profiles were mainly reflected in the overall level of humanistic practice ability. Accordingly, this study named the classes descriptively based on their overall performance. Most nurses were classified into the Moderate-Level Profile, while the proportion classified into the High-Level Profile was relatively small. Traditional variable-centered approaches mainly evaluate individuals using total scores or mean scores across dimensions. In contrast, LPA takes a person-centered approach and identifies latent subpopulations with similar response patterns. This approach can provide additional information for understanding heterogeneity within a group (29). Therefore, applying LPA in this study provides information that complements traditional total-score evaluation. This approach offers a methodological reference for more accurately characterizing within-group differences in humanistic practice ability among psychiatric nurses. It may also facilitate subsequent stratified assessments.

The results of this study showed a statistically significant association between years of working and profile membership of humanistic practice ability. Compared with nurses with ≥20 years of working, nurses with shorter years of working were less likely to be classified into the High-Level Profile in certain between-class comparisons. The previous study has similarly found differences in nurses’ humanistic practice ability across groups with different years of working (9). This association may be related, to some extent, to the accumulation of clinical experience. However, the study did not directly investigate the specific mechanisms through which accumulated clinical experience may be associated with humanistic practice ability. Accordingly, the findings do not support the inference that an increase in years of working directly enhances nurses’ communication, emotional coping, or ethical practice abilities. From a nursing practice perspective, attention could be paid to the characteristics of humanistic practice ability among nurses with different years of working, and the exchange of clinical experience and practical knowledge within teams could be promoted (30). However, the actual effectiveness of such support approaches still requires further evaluation in future research.

This study found a relatively consistent association between EI and profile membership of humanistic practice ability among psychiatric nurses, with nurses having higher levels of EI more likely to be classified into higher-level humanistic practice ability profiles. The Previous study has also suggested an association between EI and humanism-related nursing competencies (31). For example, Ma et al. used the Wong and Law Emotional Intelligence Scale to assess the EI of hospital staff, and found that EI was positively correlated with humanistic caring ability among hospital staff (32). Psychiatric nursing is characterized by strong emotional interaction, as nurses must continuously face patients’ complex emotional and behavioral presentations in their clinical work. Therefore, EI is a noteworthy individual-level factor to consider when understanding the different humanistic practice ability profiles of psychiatric nurses.

This study found a statistically significant association between nurses’ job stressors and latent profile of humanistic practice ability among psychiatric nurses. Higher levels of job stressors were associated with profiles characterized by lower levels of humanistic practice ability. A study of 257 ICU nurses by Zhang et al. similarly found a negative correlation between nurses’ job stressors and humanistic practice ability (15). In addition, Bu et al. found that nursing organizational climate was positively correlated with nurses’ humanistic practice ability, suggesting an association between work environment factors and humanistic practice ability (33).

From the perspective of the Triadic Interaction Theory, EI can be viewed as an individual-level factor, nurses’ job stressors can be viewed as an environmental-level factor, and humanistic practice ability reflects a behavioral outcome related to nursing practice. Considering the findings presented above, both the individual-level factor of EI and the environmental-level factor of job stressors were statistically associated with latent profile of humanistic practice ability. It was consistent with the basic premise of the Triadic Interaction Theory regarding the interrelationships among individual, environmental, and behavioral factors (34). This finding suggests that the understanding of humanistic practice ability among psychiatric nurses may take into account both nurses’ individual characteristics and their work environment, rather than being explained by a single factor alone. From a nursing management perspective, when assessing nurses’ humanistic practice ability, attention could be paid to their work stress status and related work environment factors. The specific work environment support measures and their effectiveness still require further evaluation in future research.

## 5 Limitations

This study has several limitations. First, this study used convenience sampling, and participants were mainly recruited from psychiatric hospitals in Jiangsu Province. Therefore, the representativeness of the sample may be somewhat limited, and caution should be exercised when generalizing the findings to other regions or different types of healthcare institutions. Second, due to the cross-sectional design of this study, the temporal ordering and causal relationships among EI, nurses’ job stressors, and latent profile of humanistic practice ability could not be established. Moreover, the dynamic interactions among individual, environmental, and behavioral factors proposed by Triadic Interaction Theory were not directly tested. Third, humanistic practice ability, EI, and nurses’ job stressors in this study were all measured using self-report scales, and thus the influence of self-report bias cannot be completely ruled out. In addition, humanistic practice ability, EI and nurses’ job stressors in this study were all measured using self-rated scales, and therefore a certain degree of self-report bias may exist. Future studies could adopt multicenter, larger-sample, and longitudinal designs, combined with multi-source or more objective data collection methods, to further validate the findings of this study and improve the representativeness and robustness of the results.

## 6 Conclusions

Using LPA, this study identified three latent profiles of humanistic practice ability among psychiatric nurses, namely the Low-Level Humanistic Practice Profile, the Moderate-Level Humanistic Practice Profile, and the High-Level Humanistic Practice Profile, indicating within-group heterogeneity in humanistic practice ability among psychiatric nurses. Years of working, EI, and nurses’ job stressors were statistically associated with membership in the different latent profiles. From a person-centered perspective, this study provides complementary information beyond traditional total-score evaluation. It also offers a reference for identifying subgroups of psychiatric nurses with distinct humanistic practice ability profiles and for conducting further stratified assessments.

## Data Availability

The raw data supporting the conclusions of this article will be made available by the authors, without undue reservation.

## References

[1] Zhang J Zhou X Wang H Luo Y Li W . Development and validation of the humanistic practice ability of nursing scale. Asian Nurs Res (Korean Soc Nurs Sci). (2021) 15:105–12. doi: 10.1016/j.anr.2020.12.003 33453453

[2] Wang Y Zhang Y Liu M Zhou L Zhang J Tao H . Research on the formation of humanistic care ability in nursing students: a structural equation approach. Nurse Educ Today. (2020) 86:104315. doi: 10.1016/j.nedt.2019.104315 31896034

[3] Ozdemir C Kaplan A . Examining nurses' humanistic behaviour ability and empathy levels in clinical practice: a descriptive and exploratory study. J Clin Nurs. (2024) 33:3115–27. doi: 10.1111/jocn.16992 38234293

[4] Aiken LH Sloane DM Bruyneel L Van den Heede K Griffiths P Busse R . Nurse staffing and education and hospital mortality in nine European countries: a retrospective observational study. Lancet. (2014) 383:1824–30. doi: 10.1016/S0140-6736(13)62631-8 24581683 PMC4035380

[5] Delgado C Roche M Fethney J Foster K . Mental health nurses' psychological well-being, mental distress, and workplace resilience: a cross-sectional survey. Int J Ment Health Nurs. (2021) 30:1234–47. doi: 10.1111/inm.12874 33913226

[6] Yu M Wang H Wu Y Zhang Q Du X Huang X . The influence of emotional intelligence on psychiatric nurses' care behavior, and the chain mediating role of compassion fatigue and perception of management. J Psychosoc Nurs Ment Health Serv. (2024) 63:1–9. doi: 10.3928/02793695-20241101-02 39508679

[7] Xu H Cao X Jin QX Wang RS Zhang YH Chen ZH . The impact of the second victim's experience and support on the career success of psychiatric nurses: the mediating effect of psychological resilience. J Nurs Manag. (2022) 30:1559–69. doi: 10.1111/jonm.13467 34435707

[8] Duarte J Pinto-Gouveia J Cruz B . Relationships between nurses' empathy, self-compassion and dimensions of professional quality of life: a cross-sectional study. Int J Nurs Stud. (2016) 60:1–11. doi: 10.1016/j.ijnurstu.2016.02.015 27297364

[9] Sahin A Tarsuslu B Yilmaz A Kuni F Durat G . Turkish psychometric characteristics of the humanistic practice ability of nursing scale: differences by education, working year, and professional satisfaction. BMC Nurs. (2024) 23:448. doi: 10.1186/s12912-024-02083-9 38951778 PMC11218182

[10] Delmas P O'Reilly L Cara C Brousseau S Weidmann J Roulet-Schwab D . Effects on nurses' quality of working life and on patients' quality of life of an educational intervention to strengthen humanistic practice among hemodialysis nurses in Switzerland: a protocol for a mixed-methods cluster randomized controlled trial. BMC Nurs. (2018) 17:47. doi: 10.1186/s12912-018-0320-0 30479561 PMC6249714

[11] Li Y Chi K Li W Sun X Li Y . Relationship between transition shock and humanistic practice ability among Chinese newly graduated nurses: mediating effect of organizational socialization. Nurse Educ Pract. (2023) 73:103842. doi: 10.1016/j.nepr.2023.103842 37984161

[12] Zhang L Li J Yang M Zhang Q Wu X Wang Q . Investigation of medical humanistic practice ability of nurses and factors affecting their practices: a questionnaire-based analysis of 1196 cases in a general hospital in Hunan Province. Nan Fang Yi Ke Da Xue Xue Bao. (2023) 43:139–44. doi: 10.12122/j.issn.1673-4254.2023.01.20 36856223 PMC9978721

[13] Kiratli D Duran S . Compassion fatigue and loneliness at work in nurses - the mediating role of resilience: a cross-sectional study. J Res Nurs. (2024) 29:290–302. doi: 10.1177/17449871241238965 39291224 PMC11403983

[14] Damien C Marion DH Deborah S Margot M . Professional identity and representations of advanced practice nurses specialising in psychiatry and mental health: a national study. Int J Ment Health Nurs. (2024) 33:714–25. doi: 10.1111/inm.13273 38240063

[15] Zhang Y Fei K Tang W Wang Z Pan C Wang Z . The mediating effect of professional benefits on nurse's work stressor and humanistic practice ability of ICU nurses. Nurs Open. (2026) 13:70449. doi: 10.1002/nop2.70449 42278000 PMC13260673

[16] Liu SJ Wang QN She J Zhang YH Xu H . Relationship between emotional intelligence and job stressors of psychiatric nurses: a multi‐centre cross‐sectional study. J Clin Nurs. (2023) 32:7730–9. doi: 10.1111/jocn.16865 37661580

[17] Li X Chang H Zhang Q Yang J Liu R Song Y . Relationship between emotional intelligence and job well-being in Chinese clinical nurses: multiple mediating effects of empathy and communication satisfaction. BMC Nurs. (2021) 20:144. doi: 10.1186/s12912-021-00658-4 34389005 PMC8361242

[18] Arpaci R Karasu F Ayar D . Exploring the link between emotional intelligence and compassionate communication in nurses: a cross-sectional study. West J Nurs Res. (2024) 46:640–7. doi: 10.1177/01939459241271904 39132747

[19] Bandura A . Social learning theory of aggression. J Commun. (1978) 28:12–29. doi: 10.1111/j.1460-2466.1978.tb01621.x 690254

[20] Wen Z Xie J Wang H . Principles, steps and procedures of latent class model. J East China Normal Univ (Educ Sci Edition). (2023) 41:1–15. doi: 10.16382/j.cnki.1000-5560.2023.01.001

[21] Tein J Coxe S Cham H . Statistical power to detect the correct number of classes in latent profile analysis. Struct Equation Model: A Multidiscip J. (2013) 20:640–57. doi: 10.1080/10705511.2013.824781 24489457 PMC3904803

[22] Nylund KL Asparouhov T Muthén BO . Deciding on the number of classes in latent class analysis and growth mixture modeling: a Monte Carlo simulation study. Struct Equation Model: A Multidiscip J. (2007) 14:535–69. doi: 10.1080/1070551070157539637339054

[23] Yan H . Research on the Construction of a Tool for Assessing Nurses' Humanistic. Southern Medical University, Guangzhou, China (2016). Master's thesis.

[24] Wong C Law KS . The effects of leader and follower emotional intelligence on performance and attitude. Leadersh Q. (2002) 13:243–74. doi: 10.1016/S1048-9843(02)00099-1

[25] Wang Y . Research on the Reliability and Validity of the Chinese Version of the Emotional Intelligence Scale. Central South University, Changsha, China (2010). Master's thesis.

[26] Wang Y Meng X . Preliminary revision of the Chinese nurses' job stressor scale. Chin J Clin Psychol. (2007) 02:129–31.

[27] Spurk D Hirschi A Wang M Valero D Kauffeld S . Latent profile analysis: a review and “how to” guide of its application within vocational behavior research. J Vocat Behav. (2020) 120:103445. doi: 10.1016/j.jvb.2020.10344542574925

[28] Marshman C Hansen A Munro I . Compassion fatigue in mental health nurses: a systematic review. J Psychiatr Ment Health Nurs. (2021) 29:529–43. doi: 10.1111/jpm.12812 34874593

[29] Ferguson SL Moore EWG Hull DM . Finding latent groups in observed data: a primer on latent profile analysis in Mplus for applied researchers. Int J Behav Dev. (2019) 44:458–68. doi: 10.1177/0165025419881721

[30] Kuo YL Lee JT Yeh MY . Intergenerational narrative learning to bridge the generation gap in humanistic care nursing education. Healthcare (Basel). (2021) 9:1291. doi: 10.3390/healthcare9101291 34682971 PMC8535847

[31] Sutil-Rodriguez E Liebana-Presa C Fernandez-Martinez E . Emotional intelligence, health, and performance in nursing students: a systematic review. J Nurs Educ. (2024) 63:686–92. doi: 10.3928/01484834-20240614-03 39388467

[32] Ma J Peng W Pan J . Investigation into the correlation between humanistic care ability and emotional intelligence of hospital staff. BMC Health Serv Res. (2022) 22:839. doi: 10.1186/s12913-022-08227-4 35773661 PMC9244559

[33] Bu M Ma H Zhai H Ma Y Xu N . Role of self‐efficacy in nursing organizational climate: a way to develop nurses' humanistic practice ability. J Nurs Manag. (2021) 30:2107–15. doi: 10.1111/jonm.13516 34798681

[34] Bandura A . Social cognitive theory: an agentic perspective. Annu Rev Psychol. (2001) 52:1. doi: 10.1146/annurev.psych.52.1.1 11148297

